# Microplanning for designing vaccination campaigns in low-resource settings: A geospatial artificial intelligence-based framework

**DOI:** 10.1016/j.vaccine.2021.09.018

**Published:** 2021-10-08

**Authors:** Thiago Augusto Hernandes Rocha, Dante Grapiuna de Almeida, Arthi Shankar Kozhumam, Núbia Cristina da Silva, Erika Bárbara Abreu Fonseca Thomaz, Rejane Christine de Sousa Queiroz, Luciano de Andrade, Catherine Staton, João Ricardo Nickenig Vissoci

**Affiliations:** a Duke Global Health Institute, Duke University, Durham, NC, United States of America; b MEDOMAI technology information systems, Belo Horizonte, Minas Gerais, Brazil; c Methods, Analytics and Technology for Health (M.A.T.H) Consortium, Belo Horizonte, Minas Gerais, Brazil; d Department of Public Health, Federal University of Maranhão, São Luís, Maranhão, Brazil; e Department of Medicine, State University of Maringá, Maringá, Paraná, Brazil.; f Division of Emergency Medicine, Department of Surgery, Duke University School of Medicine, Duke University, North Carolina, United States of America

**Keywords:** COVID-19, Coronavirus, Microplan, Vaccination, Vaccine, Health campaign

## Abstract

Existing campaign-based healthcare delivery programs used for immunization often fall short of established health coverage targets due to a lack of accurate estimates for population size and location. A microplan, an integrated set of detailed planning components, can be used to identify this information to support programs such as equitable vaccination efforts. Here, we presents a series of steps necessary to create an artificial intelligence-based framework for automated microplanning, and our pilot implementation of this analysis tool across 29 countries of the Americas. Further, we describe our processes for generating a conceptual framework, creating customized catchment areas, and estimating up-to-date populations to support microplanning for health campaigns. Through our application of the present framework, we found that 68 million individuals across the 29 countries are within 5 km of a health facility. The number of health facilities analyzed ranged from 2 in Peru to 789 in Argentina, while the total population within 5 km ranged from 1,233 in Peru to 15,304,439 in Mexico. Our results demonstrate the feasibility of using this methodological framework to support the development of customized microplans for health campaigns using open-source data in multiple countries. The pandemic is demanding an improved capacity to generate successful, efficient immunization campaigns; we believe that the steps described here can increase the automation of microplans in low resource settings.

## 1 Introduction

COVID-19 has placed an unprecedented pressure on health systems, and has created questions about the long-term efficiency and efficacy of campaign-based vaccine delivery given increasingly strained health systems globally [1]. Successful health campaigns often fall short of established health coverage targets due to inadequate population estimates [2]. The impossibility of finding where target populations are located hampers chances for achieving health campaign objectives; better, more accurate information regarding population sizes and locations allows for more favorable projected costs and impact evaluation of campaign-based interventions [3].

One consolidated approach to overcome these health campaign challenges that has been highlighted is the use of microplanning. A microplan is defined as an integrated set of components prepared to support the activities performed during a health campaign, used in the public health context [4]. There have been few innovations dedicated to improving the quality, automation, and generalizability of robust microplanning strategies dedicated to health campaigns. Evidence has demonstrated that GIS based microplans are more robust and achieve better results in terms of coverage of target populations [5], [6]. Despite the fact that GIS-based approaches have been used independently for immunization planning, [7] no efforts have made use of the geographic artificial intelligence (GeoAI) approach to support the creation of health campaign microplans. GeoAI combines methods from geographic information systems (GIS), artificial intelligence (AI), and data mining, and has been used in applications for several domains within public health and precision medicine, most recently in COVID-19 case predictions and surveillance [8].

Focusing on accurate location identification of eligible populations is crucial to not only successful campaigns but also for routine immunization. Until now, in order to gain advantage of GIS-based microplans, the use of GIS experts and costs of thousands of dollars in has been necessary [9].

Considering this, the aim of our study was to present steps to apply a GeoAI-based framework to conduct automated identification of populations eligible to be supported by microplanning for health campaigns. The framework proposed here will integrate information from satellite images, secondary data, and geostatistics into a user-friendly and accessible tool. By using the framework presented, health authorities will be able to identify the population within a defined distance from the health facilities enrolled in a specific health campaign. The identification of the number of individuals in a geographic area, as well as demographics including age and gender, can be used to support the development of automated GIS-based microplans. Our presented approach, implemented and tested through the series of steps listed in this paper, can contribute to the creation of a framework for campaign-based health delivery schemes as well as routine immunization.

## 2 Methods

### 2.1 Overview

We developed a sequence of methodological steps to support the creation of automated GeoAI-based population estimates for health campaign microplans. Our aim is to address major challenges, including accurately estimating up-to-date target populations and their locations, to support the creation of effective microplans. The steps listed here can be adapted to generate custom microplans using open-source databases without need for GIS experts. Additionally, the steps presented here can be applied in any country or region. The steps are generation of a conceptual framework, creation of customized catchment areas using the real-world transportation network, and estimation of the target population within each catchment area created

### 2.2 Conceptual framework

A microplan contains technical details and can be adapted as needed to fill the needs of each administrative level, whether by national institutions or health-care workers. It must work with the health service at the operational level, usually the health center, and the details of its implementation must consider the real situation of the people in field operations. To develop effective microplan frameworks, three groups of data are necessary: population distributions, locations of health facilities performing health campaign activities, and resource estimates based on the populations linked to each facility.

#### 2.2.1 Population distribution

**Fig. 1:**
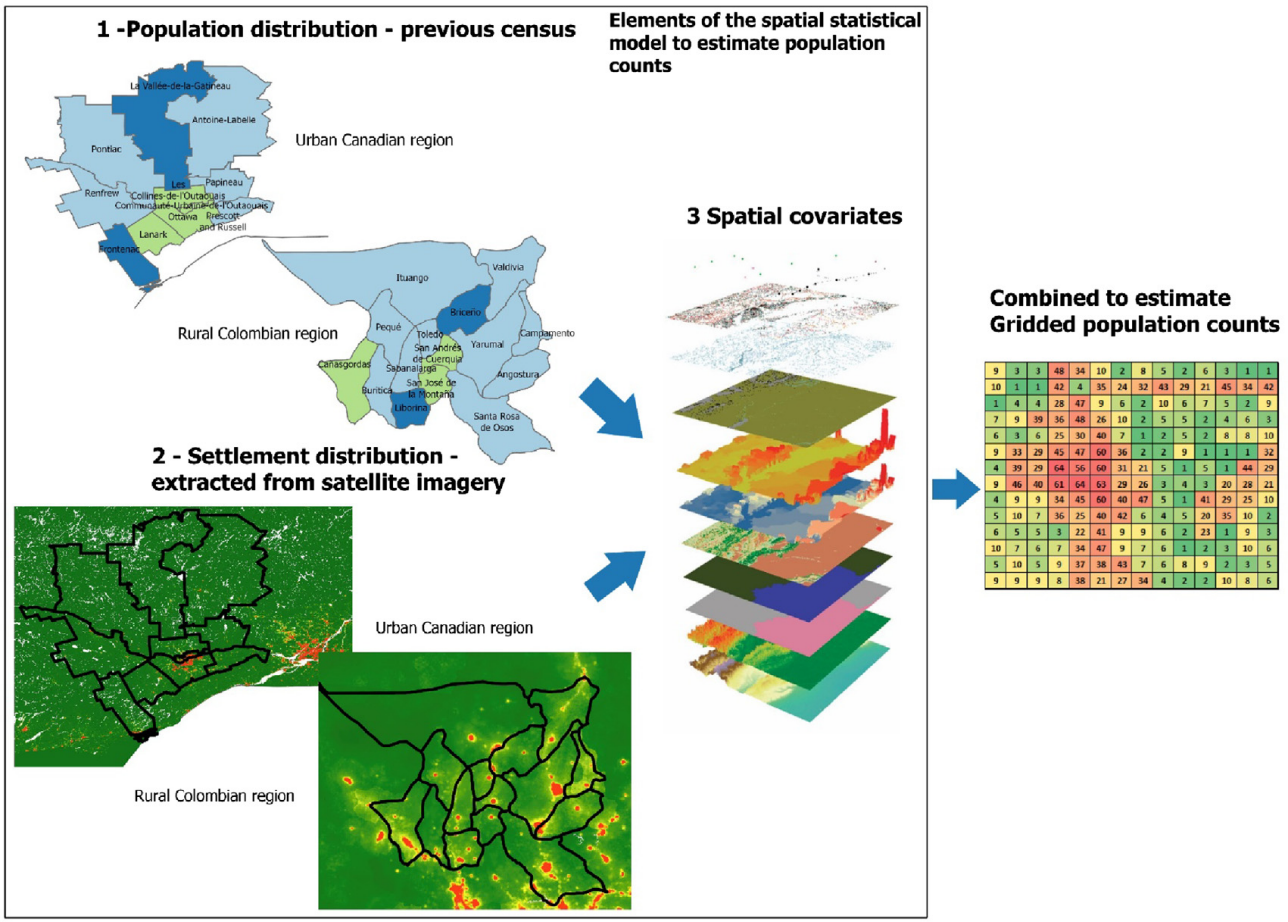
Steps and data sources used to create gridded population datasets.

Traditional microplanning efforts rely on census estimates to define the population to be addressed by a specific health campaign. However, many countries in Africa, America and Asia did not perform a census in the last 11 years [10], and the COVID-19 pandemic will continue to delay any efforts regarding a census update for at least 1–2 more years. To overcome the lack of up-to-date data concerning population distributions, recent advancements in GeoAI have supported the creation of granular population estimates worldwide. These initiatives are using gridded population datasets. Gridded population sources represent the population residing in one specific area following a gridded representation. Gridded (or raster) population maps represent the distribution of population in rows and columns of grid cells, typically defined by their latitude-longitude coordinates [11]. Thus, for every pixel covering an area or region there is number associated with it that represents the count of population in that area. These counts are obtained by applying artificial intelligence algorithms to spatial variables, and data from previous census and satellite imagery. In Fig. 1 we can see how different data sources can be combined to create a gridded dataset [12], [13].

Fig. 1 - Representation of a gridded population dataset. Source: Spatial covariates image [13].

An increasing number of data providers are combining census information with satellite-derived geospatial features to redistribute populations and produce these gridded population datasets [11]. The main advantage of this approach relies on the possibility to use recent satellite derived data as an input estimate for population count in a specific area. Thus, the estimates obtained using this approach are more reliable than outdated census data gathered years ago. The use of satellite imagery combined with spatial covariates to estimate the population counts is known as a dasymetric population forecast [14]. Currently, there are seven datasets of gridded populations based dasymetric approach, shown in Table 1.

**Table 1:** Information on dasymetric datasets.

Dasymetric population database	Description
GRIDDED POPULATION OF THE WORLD (GPW) V4	GPW models the distribution of human population counts and densities, via census data, on a continuous global raster surface (11).
GLOBAL RURAL URBAN MAPPING PROJECT (GRUMP)	GRUMP builds upon GPW as well as uses observed light data to identify urban areas (12).
GLOBAL HUMAN SETTLEMENT LAYER - POPULATION (GHS-POP)	GHS-POP depicts the distribution of population, expressed as the number of people per cell (13).
WORLD POPULATION ESTIMATE (WPE)	WPE combines information from datasets on global land cover, roads, as well as census data to calculate residential populations (14).
HIGH RESOLUTION SETTLEMENT LAYER (HRSL)	HRSL produces estimates of population distribution via census data and high-resolution satellite imagery (15).
LANDSCAN	LANDSCAN models average population locations over a 24 h period (16).
WORLDPOP	WorldPop produces a dasymetric population using Artificial Intelligence stratified by gender and age (17).

For health campaigns demanding data stratified by age and gender, the best available source is WorldPop [15]. WorldPop is the only source using artificial intelligence to redistribute census level information combined with spatial covariates and satellite imagery to perform a gridded population estimation [12]. Gridded population estimates can be done using up-to-date satellite imagery and spatial covariates data with a delay of up to only 15 days, as compared to the 10-year-old data frequently encountered in census surveys. The correct geolocation of remote populations can help planning efforts in terms of forecasting the resources needed to conduct effective health campaigns. The knowledge regarding where the target population is located, as well a close estimate of the number of people within a region, is the first information needed to structure health campaigns. From these numbers, it is possible to calculate the human resources necessary to reach the population, the displacements needed, supplies and time to cover the population to be reached.

To assess the feasibility of using population estimates obtained from satellite imagery to support health campaign microplans, we performed a pilot study across 29 countries in regions of the Americas: Antigua and Barbuda, Argentina, Barbados, Belize, Bolivia, Brazil, Canada, Chile, Colombia, Costa Rica, Cuba, Dominican Republic, Ecuador, El Salvador, Guatemala, Guyana, Haiti, Honduras, Jamaica, Mexico, Nicaraguá, Panama, Paraguay, Peru, Suriname, The Bahamas, United States, Uruguay, and Venezuela. The gridded dataset used was from WorldPop. For this work we selected the data regarding 2020, adjusted for the United Nations-provided population sizes.

To effectively link populations estimates in these American countries to their closest health facility, it is necessary to create a facility-customized catchment area. The catchment area represents a polygon around the health facility delimiting a distance in terms of meters representing the time to reach. To be able to create these polygons and analyze the time or distance to be travelled to reach the health campaign points, the geolocation of facilities is necessary.

#### 2.2.2 Geolocation of health facilities

Often, local governments have the addresses of existing health facilities in a country. An address itself, however, does not contain latitude and longitude coordinates. To overcome such challenges, the OpenStreetMap [21] initiative offers an open data source updated on a yearly basis that covers 246 countries and territories. From the OpenStreetMap database there are Application Programming Interfaces (API) allowing the conversion of text addresses to latitude and longitude coordinates. Examples of such APIs are Opencage and Mapquest [16], [17]. Considering the value per query and the geocoding limits, the OpenCage API offers the best available cost-benefit relation and parallelization capabilities. Additionally, the OpenCage API offers a ready-to-use Software Development Kit in 30 programming languages, including R and Python [18], [19].

Despite this, there are global initiatives aiming to address the health facility geocoding challenge. Healthsites.io [20] is the first attempt to build a global open data source master list of health facilities. As of August 2021, there are 906,403 points registered in more than 120 countries. To demonstrate the feasibility of the suggested approach, we analyzed data from 5,424 hospitals and clinics in the 29 selected countries. Each facility was considered as a potential health campaign point of care. Around each one, we developed an approach to create custom defined catchment areas. These areas were used to calculate the amount of population located within their limits, allowing us to estimate the campaign resources needed as well as the logistics plans to reach the target population and underserved areas. The broad variability in terms of land cover, street distribution, and transportation network contributes to the complexity involved in the creation of catchment areas, however the ability to create custom-made catchment areas based on actual transportation networks is essential to support the definition of regions reflecting the actual dynamic of population flow.

### 2.3 Development of an approach to create customized catchment areas in healthcare domains

#### 2.3.1 Combining ArcGIS service area methodology and OpenStreetMap to create customized catchment areas for each health facility across the globe

The creation of polygons over the transportation network reflects the possible routes to be taken by the population attempting to reach a health facility. Usually, the creation of catchment areas is done considering straight line displacements. The use of this approach is not reliable, as a straight-line displacement not always represents the real possibilities of displacement. Instead of using the straight-line approach, we developed a tool to create catchment areas considering the real displacement possibilities over the actual transportation network existing, like roads, rivers, ferry lines, railways, and on foot pathways [21]. By improving the way to create catchment area we leveraged the quality of the catchment areas used to estimate the population close to a vaccination point. Additionally, the polygons created over the actual mobility network can take into consideration transportation modes as walking, automobile, or public transportation.

There are several implemented GIS routines capable of generating catchment areas over a transportation network [22]. The present work used the ArcGIS Pro [23] service area approach due to its integration with Python scripting language. The transportation dataset used came from OpenStreetMap. To assess transportation mode, we used walking distance and defined the threshold distance to reach the health facility as five kilometers. In total, 5,424 catchment areas were created in the 29 countries selected. The approach developed by us can be used in any country of the globe to create catchment areas using different displacement limits in terms of time or distance defined by the end-user.

#### 2.3.2 Spatial overlapping as an approach to identify target population and underserved areas

Once polygons characterizing the time or the distance needed to reach health facilities have been defined, it is possible to overlap them with the gridded population estimates. The population outside the catchment areas created can be considered as being in underserved areas. We selected every one of the 29 countries and chose two regions within each to be analyzed. The first region was an area close to the country capital, to investigate the performance of the approach within an urban area. The second region was a rural area of the country with availability of at least one health facility. By using two regions, we believe that it is possible to test the feasibility of using our proposed approach in both urban and rural multi-country contexts.

Vaccination campaigns usually need to address specific age groups within a population, and the WorldPop gridded dataset allows us to better prioritize interventions for sub-populations stratified by age. By estimating the volume of population within the catchment area of the 5,424 analyzed health facilities, it is possible to compute the necessary amount of resources needed to offer a vaccination campaign.

### 2.4 Microplan and rapid assessment tools

#### 2.4.1 Creation of customized microplans from the population burden estimated through the innovative approach suggested

Usually, a microplan is composed of six sections: resource estimation, cold-chain logistics, operations, supervision, recording and reporting tools, and monitoring framework [24], [25]. The details of its implementation must consider the real situation of the people in field operations; otherwise, the microplan will fail to accomplish its objectives. Flexibility to make changes to suit local conditions must be possible at every step [4]. The ability to adjust the catchment area is crucial to creating microplans tailored to the changing circumstances present in field operations. We opted to use open data sources to validate the polygons created with no need for GIS or programming skills. Thus, health professionals can validate with local data the polygons created to best reflect the field challenges. The population estimates can be used to plan the six core elements of a standard microplan.

## 3 Results

Table 2 describes the population within 5 km of walking distance to each health facilities analyzed. The number of health facilities analyzed ranged from two in Peru to 789 in Argentina, while the total population within five km ranged from 1,233 in Peru to 15,304,439 in Mexico. The average number of people covered by each facility was higher in North America than in the other portions of the continents. Our results show that, considering a five km distance for the selected facilities, it is possible to cover 68 million people across the 29 assessed countries.

**Table 2:** Results of pilot study across 29 American countries.

Continent/Country	Health facilities (N)	Total population covered	Average population by facility	Standard deviation	Population by facility (Minimum)	Population by facility (Maximum)
Central America	1,789	15,232,520	8,515	13,264	1	155,785
Antigua and Barbuda	8	42,404	5,301	4,584	48	14,246
Barbados	11	74,168	6,743	5,100	38	16,583
Belize	8	19,233	2,404	2,420	73	7,557
Costa Rica	282	2,013,012	7,138	9,630	1	83,089
Cuba	303	1,255,638	4,144	6,556	5	45,055
Dominican Republic	147	1,829,897	12,448	14,475	41	86,348
El Salvador	98	1,005,075	10,256	14,847	33	90,269
Guatemala	142	1,761,734	12,407	24,425	31	155,785
Haiti	459	3,438,237	7,491	9,944	2	54,210
Honduras	70	1,005,566	14,365	18,939	33	80,296
Jamaica	41	721,968	17,609	18,043	20	70,585
Nicaragua	149	1,104,973	7,416	11,566	20	59,468
Panama	63	826,463	13,118	15,560	30	83,284
The Bahamas	8	134,152	16,769	13,869	65	33,677
North America	1,127	22,654,898	20,102	40,410	0	585,692
Canada	450	2,511,960	5,582	9,433	0	77,191
Mexico	403	15,304,439	37,976	60,889	0	585,692
United States	274	4,838,499	17,659	17,445	0	565,826
South America	2,508	30,862,644	12,306	25,729	0	565,826
Argentina	789	9,189,609	11,647	12,009	10	73,459
Bolivia	262	1,412,150	5,390	12,307	23	116,954
Brazil	265	3,348,960	12,638	16,577	0	82,663
Chile	166	2,452,218	14,772	22,136	14	142,714
Colombia	264	9,176,983	34,761	64,458	3	565,826
Ecuador	437	2,015,988	4,613	7,299	1	77,766
Guyana	29	162,454	5,602	5,729	0	20,210
Paraguay	71	534,709	7,531	7,362	69	36,576
Peru	2	88,103	4,405	4,919	3	17,640
Suriname	20	88,103	4,405	4,919	3	17,640
Uruguay	91	1,441,047	15,836	19,022	46	109,569
Venezuela	112	1,039,190	9,278	13,363	4	74,548
Total	5,424	68,750,062	12,675	26,839	0	585,692

While the units gathered through the healthsites.io application represents only a sample of the actual units of every location our approach can be applied to calculate the amount of population close to each health service. As the methodology we developed depends on the current number of facilities as well as their location, any change in the number of health services available or in its location can change the amount of population linked to each facility. Changes regarding these will impact the microplan’s development in terms of the numbers of resources needed.

**Fig. 2:**
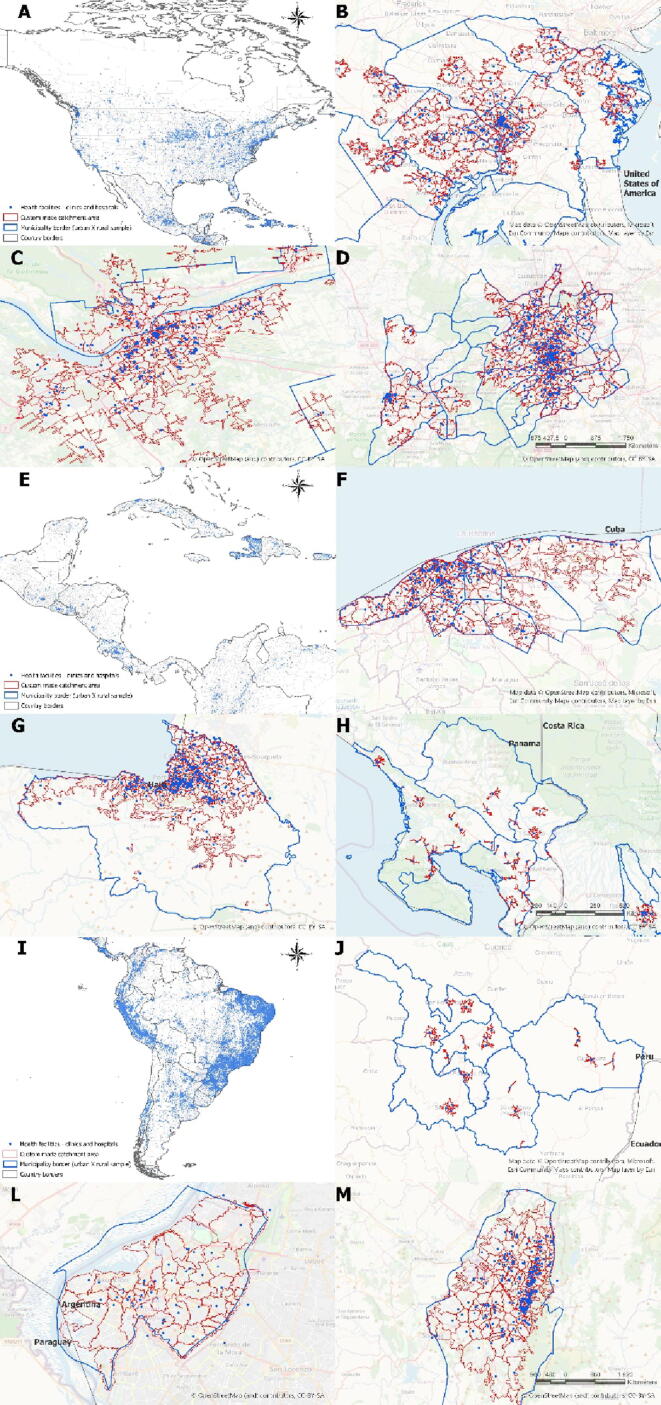
Health facilities and respective catchment populations in Americas. A North America distribution of facilities and service areas. B EUA, Washington D.C area. C Canada, Toronto area. D Mexico, rural Mexico region. E Central America distribution of facilities and service areas. F Cuba, Havana. G Haiti, Porto Principe. H Costa Rica, rural area. I South America distribution of facilities and service areas. J Ecuador, rural area. L Paraguay, Asuncion. M Colombia, Bogotá.

**Fig. 3:**
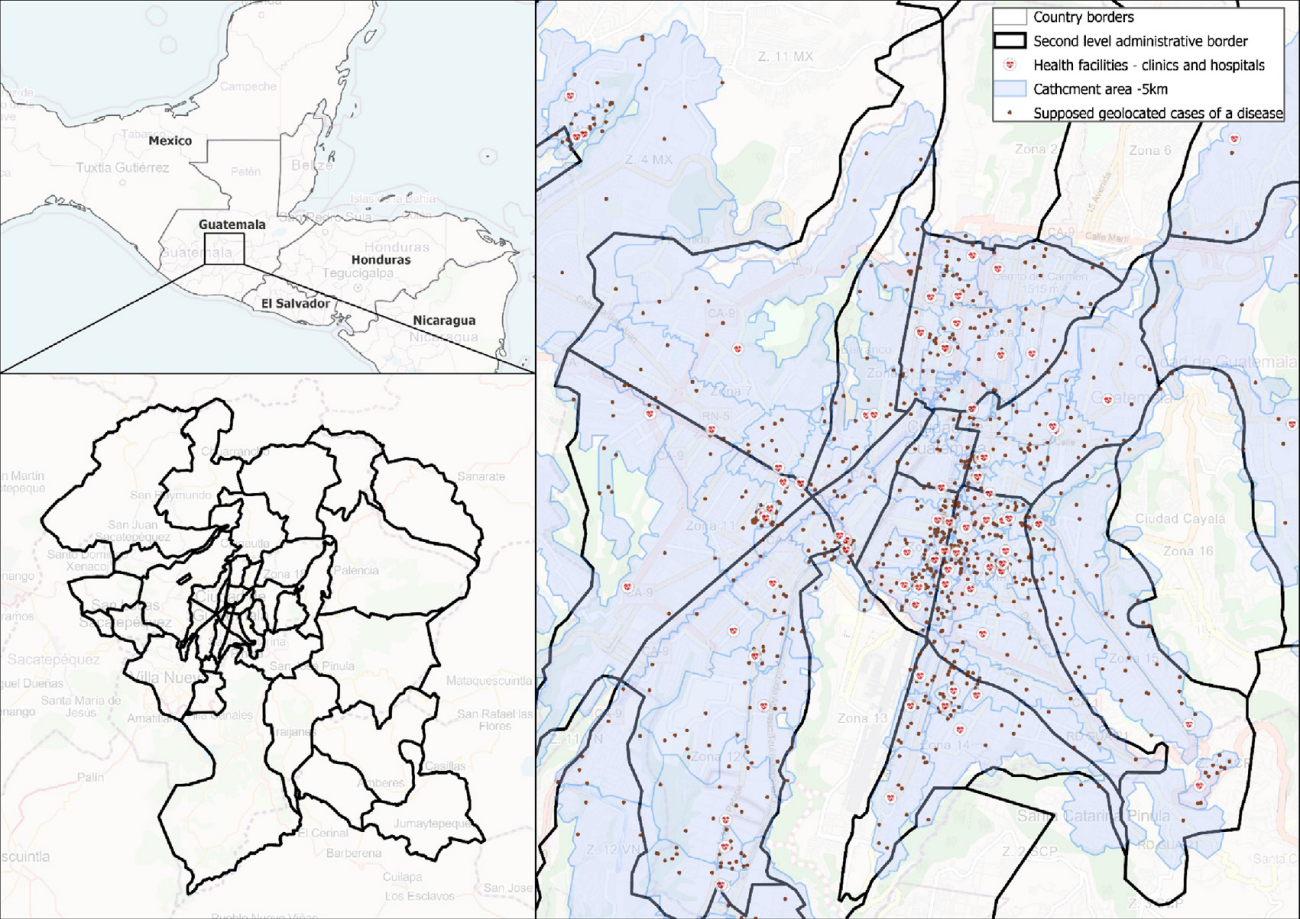
Example of how the present solution can support the development of GIS-based microplanning.

Fig. 2 represent, for parts of the America continents, the spatial distribution of the facilities analyzed, as well as the service areas created to estimate the burden of population by each health service. For every country, we highlighted both the rural area and region close to the country capital. An interactive version of the following maps can be found here: Fig. 3 represents random points within the catchment areas created. These points could represent cases of a disease being monitored. Considering the volume of population estimated in each catchment area, it is possible to calculate vaccine coverage, rates of cases per population, and where there is a disease presence. Thus, by using this type of information it is possible to better drive the microplanning of health campaign interventions or routine immunization actions.

## 4 Discussion

Our results demonstrate the feasibility of using our described methodology as an effective approach to support the identification of eligible populations to customize microplans for health campaigns. Using open sources, we were able to estimate the populations close to health facilities, necessary to design a vaccination plan capable of identifying underserved areas. The location of difficult-to-reach settlements was estimated from satellite imagery of 2020, overcoming the challenge imposed by outdated census surveys. Additionally, our results demonstrated that the proposed methodology can be applied in a multi-country context, as we were capable of estimating the burden of population by health facility to 29 countries.

Population location and size are the main drivers of an effective microplan design. Supported by a gridded population stratified by age and gender, policymakers will be capable of designing, for example, COVID-19 vaccination plans that prioritize regions according to an epidemiological profile of higher risk. The methodology we developed can help to better drive the COVID-19 vaccination resources, optimizing the deployment of the vaccine to the areas where it can contribute to diminish the volume of acute COVID-19 manifestation.

The mere existence of a vaccine does not assure that it will reach the target population: quality planning is a key enabler of effective campaign implementation and is critical to support campaign performance. A microplan, which specifically addresses the detailed, delivery-level planning required to reach intended populations with a health intervention, is recognized as a critical driver of campaign success [24]. Our approach can assist health campaigns in achieving higher coverage of target populations, better identifying and reaching high-risk/unreached populations, and more efficiently using resources.

An effective microplan depends on precise information from the point of care perspective. The solution currently available to create GIS-supported microplans does not contain features to integrate information from the field, and efforts to adapt currently available platforms and approaches cost thousands or even millions of dollars [9]. Our solution, in contrast, can be integrated with open data sources platforms to incorporate data from the field to reshape the service areas built, using platforms based on OpenStreetMap such as uMap [26]. Using uMap, health planners can create health facility service areas and share this information for the professionals in the field. The end-user, without any expert knowledge of GIS, can reshape the service area previously drawn to reflect a more precise coverage area, taking into consideration data from the field. To leverage the possibilities of applying the steps described in this manuscript we are releasing an ArcGIS toolbox used to run the analysis described through this work.

Another crucial feature to qualify the data from health campaigns, specially vaccination campaigns, is the possibility to perform rapid monitoring assessments. Through tools such as Open Data Kit (ODK) [27], it is possible to create custom made electronic surveys to gather data from campaign progress, geolocation of cases, adverse and associated effects of vaccination, even considering circumstances where an internet connection is not available. By integrating a tool to support collaborative mapping, such as uMap and ODK, with the methodological steps of the present manuscript, it is possible to integrate an ecosystem capable of fostering the design of an effective microplan for any country or region in the world.

The supplementary material of this manuscript provides an ArcGIS toolbox that can be loaded to ArcGIS to run all the analytical steps discussed with a few clicks, without the need of knowledge regarding coding skills (https://doi.org/10.6084/m9.figshare.13908209.v1). Thus, any intermediate GIS user would be capable of replicating the steps defined by our methodology.

## 5 Limitations and future research

When data from WorldPop was compared with local data collected, some differences may be observed. Healthsites.io offers the location of health facilities across the globe but does not provide a full description of all existing health facilities. Although such limitations regarding the approach developed exist, the use of these data sources are the best available option to handle a global challenge.

The contribution provided by our approach helps to reduce the need for multiple experts in AI, GIS and remote health sensing experts, but the best solution to scaling-up the use of GIS supported microplans would be tailored to end-users without an intermediate knowledge of GIS. This type of method needs a cloud computing solution, capable of integrating each part of the solution developed, and could be supported by international non-governmental organizations, or countries' Ministry of Health. Embedding the methodological steps described through the manuscript in a cloud solution can help to integrate the different tools used in just one solution.

## Declaration of Competing Interest

The authors declare that they have no known competing financial interests or personal relationships that could have appeared to influence the work reported in this paper.
